# Supplementary material for: Tyrosine transfer RNA levels and modifications during blood‐feeding and vitellogenesis in the mosquito, Aedes aegypti
Source: Insect Mol Biol. 2024 Aug 6;34(1):65–80. doi: 10.1111/imb.12950 (PMC11705514; doi:10.1111/imb.12950)
Supplement: Supplementary file 1 — Table S1. Genes of tRNA‐modifying enzymes identified in A. aegypti. The list of previously identified genes in A. aegypti associated with tRNA modification. The microarray IDs from Dissanayake et al 2010 are also listed. Table S2. MS parameters for SRM detection of nucleosides in the mosquito fat body. Table S3. All combinations of interactions between tRNA modification, modifying enzyme expression, time, and blood‐feeding. Table S4. PerMANOVA results for determining tRNA‐modifying enzymes that exhibit altered expression in response to blood‐feeding. Table S5. PerMANOVA results for determining modifications affected by blood‐ feeding. Figure S1. Agarose gel confirming the presence of pure tRNA prior to nucleoside digestion. In each gel, the first lane on the left is a yeast tRNA standard to demonstrate the band size is approximately the same for each tRNA sample (~100bp) and the absence of contaminating RNA (i.e., ribosomal RNA bands) or salt contamination. The other lanes are tRNA samples isolated from A. aegypti mosquitoes in the hours following a bloodmeal. The hour of collection is indicated first and the biological replicate number following. The bands appear dark due to high amounts of sample, causing saturation during imaging. A. Order (left to right): tRNA standard, NBF1, NBF2, NBF3, 72H1, 72H2, 72H3. B. Order (left to right): tRNA standard, 6H1, 6H2, 6H3, 12H1, 12H2, 12H3. C. Order (left to right): tRNA standard, 24H1, 24H2, 24H3, 48H1, 48H2, 48H3. Figure S2. Relative synonymous codon usage (RSCU) of tyrosine codons indicates UAC is the preferred codon in Dipteran species. A. An RSCU value >1.5 indicates preference for a codon. A codon is considered unpreferred is the RSCU <0.5. Despite the same preferred codon, the codon UAU is unpreferred in ribosomal proteins and vitellogenin. B. Upon further investigation, UAC is the preferred codon in vitellogenin transcripts of other mosquito species and Drosophila melanogaster, another Dipteran species. However, this [file IMB-34-65-s001.pdf]

Supplemental Figures and Tables

Supplemental Table 1. Genes of tRNA-modifying enzymes identified in *A. aegypti*. The list of previously identified genes in *A. aegypti* associated with tRNA modification. The microarray IDs from Dissanayake et al 2010 are also listed.

Gene	Name	Modification	ID_1	ID_2
AAEL007354	PUS1	Ψ	12230	32222
AAEL004663	PUS3	Ψ	19066	41711
AAEL003071	PUS7	Ψ	14193	17473
AAEL010362	PUS10	Ψ	35068	36380
AAEL005898	DUS2	D	671	42043
AAEL001171	DUS3L	D	5550	1.09E+04
AAEL008120	FTSJ1A	Cm/Gm	14814	30069
AAEL001037	FTSJ1B	Cm/Gm	30196	31442
AAEL014096	TRMT44	Um	10629	31124
AAEL011199	TRMT13	Cm/Am	11230	29865
AAEL003922	TRMT11	m^2G	5108	8488
AAEL012932	METTL6	m^3C	10058	37114
AAEL007084	METTL2B	m^3C	22856	43453
AAEL009603	TRMT5	m^1G	18667	22767
AAEL008124	TRMT61A1	m^1A	5632	19910
AAEL003654	TRMT61A2	m^1A	11245	32926
AAEL008941	TRMT10A	m^1A	4156	34445
AAEL011538	TRMT10C	m^1G/m^1A	5938	11440
AAEL006166	TRDMT1	m^5C	17034	26850
AAEL012520	NSUN3	m^5C	34636	39482
AAEL010349	TRMT1	$m^2{}_2G$	7195	33365
AAEL004625	DTWD1	acp^3U	9592	30264
AAEL010993	QTRT1	Q	9669	17759

AAEL010968	QTRT2	Q	8566	18414
AAEL001036	ELP1	mcm^5U	7937	27473
AAEL004333	ELP4	mcm^5U	10072	19052
AAEL004571	OSGEPL1	t^6A	22530	33617
AAEL006221	TP53RK	t^6A	2144	40735
AAEL007313	TrmO	m^6t^6A	16015	32419
AAEL002837	CDK5RAP1	ms^2i^6A	13433	27734

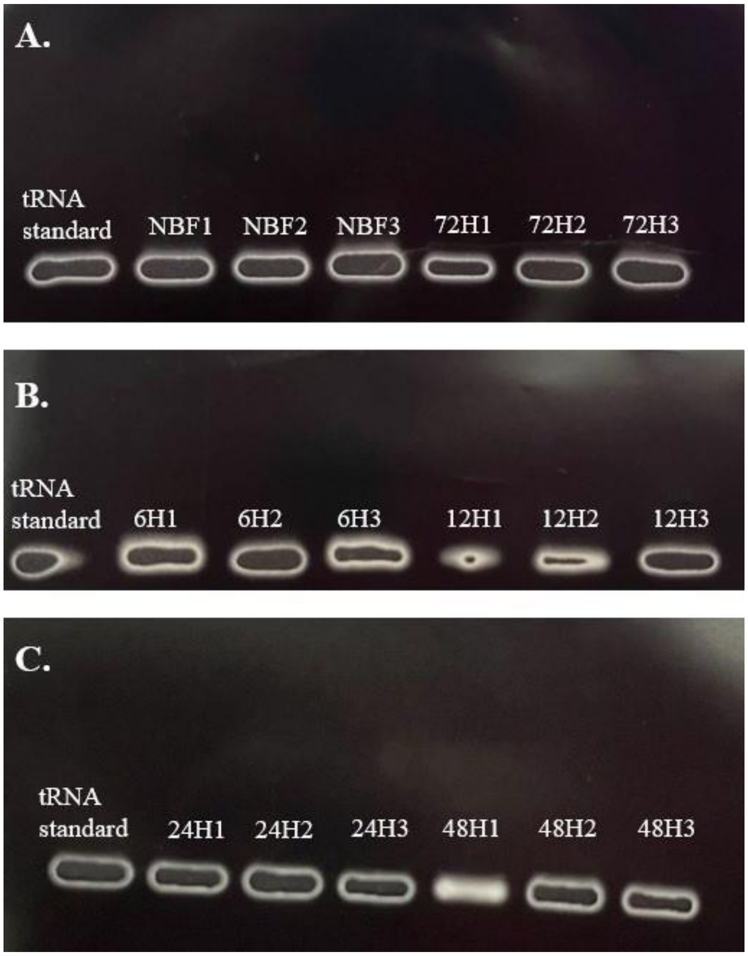

Supplemental Figure 1. Agarose gel confirming the presence of pure tRNA prior to nucleoside digestion. In each gel, the first lane on the left is a yeast tRNA standard to demonstrate the band size is approximately the same for each tRNA sample (~100bp) and the absence of contaminating RNA (i.e., ribosomal RNA bands) or salt contamination. The other lanes are tRNA samples isolated from *A. aegypti* mosquitoes in the hours following a bloodmeal.

The hour of collection is indicated first and the biological replicate number following. The bands appear dark due to high amounts of sample, causing saturation during imaging. **A.** Order (left to right): tRNA standard, NBF1, NBF2, NBF3, 72H1, 72H2, 72H3. **B.** Order (left to right): tRNA standard, 6H1, 6H2, 6H3, 12H1, 12H2, 12H3. **C.** Order (left to right): tRNA standard, 24H1, 24H2, 24H3, 48H1, 48H2, 48H3.

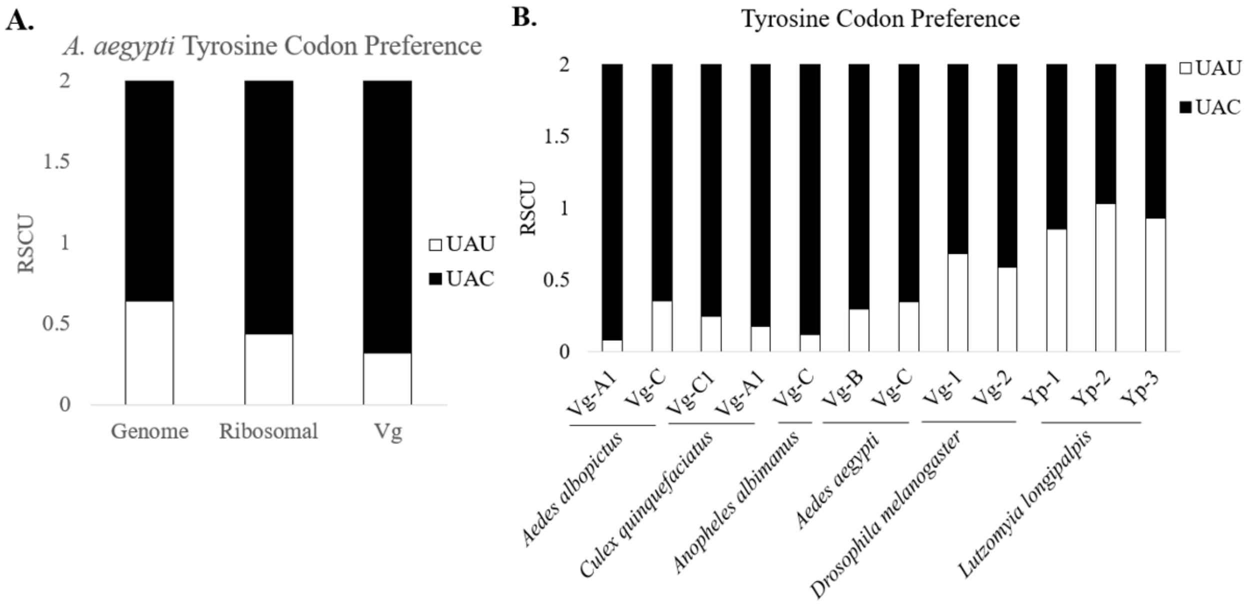

Supplemental Figure 2. Relative synonymous codon usage (RSCU) of tyrosine codons indicates UAC is the preferred codon in Dipteran species. A. A RSCU value > 1.5 indicates preference for a codon. A codon is considered unpreferred is the RSCU < 0.5. Despite the same preferred codon, the codon UAU is unpreferred in ribosomal proteins and vitellogenin. **B.** Upon further investigation, UAC is the preferred codon in vitellogenin transcripts of other mosquito species and *Drosophila melanogaster*, another Dipteran species. However, this trend does not apply to the sand fly, Lutzomyia longipalpis; demonstrating codon usage preferences in Vg transcripts are not necessarily uniform across Diptera.

Supplemental Table 2. MS parameters for SRM detection of nucleosides in the mosquito fat body.

Compound	Retention Time (min)	Precursor (m/z)	Product (m/z)	Collision Energy (V)	RF Lens (V)
Ψ	2	245	209	10	30
C	3	244	112	12	41
D	3	247	115	10	43
m^1A	3	282	150	20	74
U	3.35	245	113	10	30
acp^3U	3.71	346	214	10	50
acp^3D	2.8	348	216	10	50

m^3C	4.07	258	126	14	46
m^6A	5.17	282	150	20	74
m^5C	5.61	258	126	14	46
Ψm	5.83	259	223	10	30
Cm	7.89	258	112	12	41
I	9.23	269	137	10	40
m^7G	9.37	298	166	16	50
m^5U	9.55	259	127	10	30
G	11	284	152	14	46
m^3U	13.27	259	127	10	30
Um	14.17	259	113	10	30
Um	14.17	259	127	10	30
oQ	15.21	426	163	29	84
oQ	15.21	426	295	12	84
Q	17.6	410	295	16	49
Q	17.6	410	163	31	96
ISTD	18.7	613	306	12	41
Gm	20.94	298	152	11	50
mcm^5U	21	317	125	10	30
mcm^5U	21	317	153	10	30
mcm^5U	21	317	185	10	30
m^1I	21.97	283	151	20	74
manQ	22.94	572	163	35	96
manQ	22.94	572	295	35	96
ac^4C	24.16	286	154	10	46
A	24.21	268	136	17	63
m^1G	28	298	166	16	50
m^2G	30	298	166	16	50
$m^2{}_2G$	31.2	312	180	15	60
m^6t^6A	33.25	427	295	31	96
Am	33.39	282	136	17	63
mcm^5s^2U	34.06	333	141	35	96
mcm^5s^2U	34.06	333	169	35	96
mcm^5s^2U	34.06	333	201	35	96
ms^2t^6A	36.04	459	208	31	96
ms^2t^6A	36.04	459	327	31	96
i^6A	36.54	336	204	31	96
i^6A	38.39	336	148	31	96
i^6A	38.39	336	204	31	96
i^6A	38.39	336	136	31	96
ms^2i^6A	40.93	382	250	31	96
t^6A	34.75	413	281	31	96

Supplemental Table 3. All combinations of interactions between tRNA modification, modifying enzyme expression, time, and blood-feeding. Statistics generated from

Modification	Gene	Treatment F-statistic	P-value	Time F-statistic	P-value
Ψ	PUS1	70.395	5.38E-07	6.882	0.0001
	PUS3	0.87	0.445	1.8	0.125
	PUS7	18.03	0.0003	6.871	0.0001
D	DUS2	9.57	0.003	6.1	0.0002
	DUS3L	5.46	0.02	4.85	0.001
m^3C	METTL6	7.34	0.009	6.24	0.0002
	METTL2B	1.9	0.195	5.8	0.0003
m^5C	TRDMT1	1.88	0.198	1.45	0.226
	NSUN3	0.233	0.795	1.77	0.131
Cm	FTSJ1A	0.285	0.756	2.42	0.044
	FTSJ1B	6.8	0.01	4.98	0.001
	TRMT13	0.15	0.862	2.99	0.01
Um	TRMT44	0.458	0.644	2.11	0.074
m^1A	TRMT10C	7.02	0.01	2.86	0.021
	TRMT10A	0.639	0.546	2.88	0.02
m^2G	TRMT11	1.63	0.239	3.14	0.01
Gm	FTSJ1A	1.83	0.205	1.32	0.278
	FTSJ1B	20.37	0.0001	2.82	0.023
m^1G	TRMT10C	36.96	1.31E-05	5.32	0.0006
	TRMT10A	21.86	0.0001	4.97	0.001
m^2_2G	TRMT1	2.4	0.136	1.72	0.144
mcm^5U	ELP1	0.816	0.467	1.92	0.102
	ELP4	1.01	0.395	1.94	0.1
acp^3U	DTWD1	0.609	0.561	2.05	0.082
Q	QTRT1	0.037	0.963	1.92	0.102
	QTRT2	0.379	0.693	4.564	0.001
t^6A	OSGEPL1	12.223	0.001	1.331	0.275
	TP53RK	15.54	0.0006	1.59	0.179
ms^2i^6A	CDK5RAP1	0.674	0.529	1.78	0.13
m^6t^6A	TRMO	0.652	0.54	1.609	0.174

Supplemental Table 4. PerMANOVA results for determining tRNA-modifying enzymes that exhibit altered expression in response to blood-feeding.

Enzyme	F-statistic	P-value
ELP1	1.169	0.3791
ELP4	0.5882	0.7094
DTWD1	0.4563	0.8012
OSGEPL1	2.001	0.1507
TP53RK	5.721	0.00631

TrmO	0.9174	0.5021
TRMT1	1.64	0.2233
TRMT44	1.264	0.3405
TRMT10C	6.244	0.00447
TRMT5	6.714	0.00333
TRMT61A1	8.113	0.0015
TRMT61A2	4.784	0.01231
TRMT10A	3.766	0.02779
TRMT13	7.667	0.00191
DUS3L	9.238	0.00084
DUS2	16.27	5.55E-05
METTL6	343.2	1.63E-12
METTL2B	11.08	0.00037
NSUN3	1.681	0.2136
TRDMT1	2.407	0.09866
FTJS1A	2.702	0.07353
FTJS1B	34.71	9.84E-07
PUS1	44.18	2.56E-07
PUS3	1.934	0.162
PUS7	21.82	1.21E-05
PUS10	2.047	0.1436
CDK5RAP1	1.227	0.3551
QTRT1	0.3359	0.8816
QTRT2	4.227	0.01898

Supplemental Table 5. PerMANOVA results for determining modifications affected by blood-feeding.

Modification	F-statistic	P-value
Ψ	3.263	0.043
D	2.774	0.068
m^3C	2.918	0.059
m^5C	1.238	0.35
Cm	1.46	0.272
Um	2.614	0.08
m^3U	2.614	0.08
I	1.293	0.329
Am	1.017	0.449
m^1A	1.742	0.199
m^2G	1.714	0.205
Gm	1.047	0.434
m^1G	13.17	0.0001
$m^2{}_2G$	1.739	0.2003

mcm^5U	5.959	0.005
i^6A	4.069	0.021
acp^3U	4.81	0.012
Q	5.206	0.009
t^6A	4.254	0.018
ms^2i^6A	5.57	0.006
m^6t^6A	2.784	0.067
ms^2t^6A	7.319	0.002
m^7G	2.052	0.142
acp^3D	1.079	0.419
m^5U	1.193	0.368
Ψm	4.216	0.019
m^6A	2.735	0.071
m^1I	2.507	0.089
ac^4C	1.305	0.325
m^2G	1.714	0.205
oQ	7.607	0.001
manQ	6.599	0.003

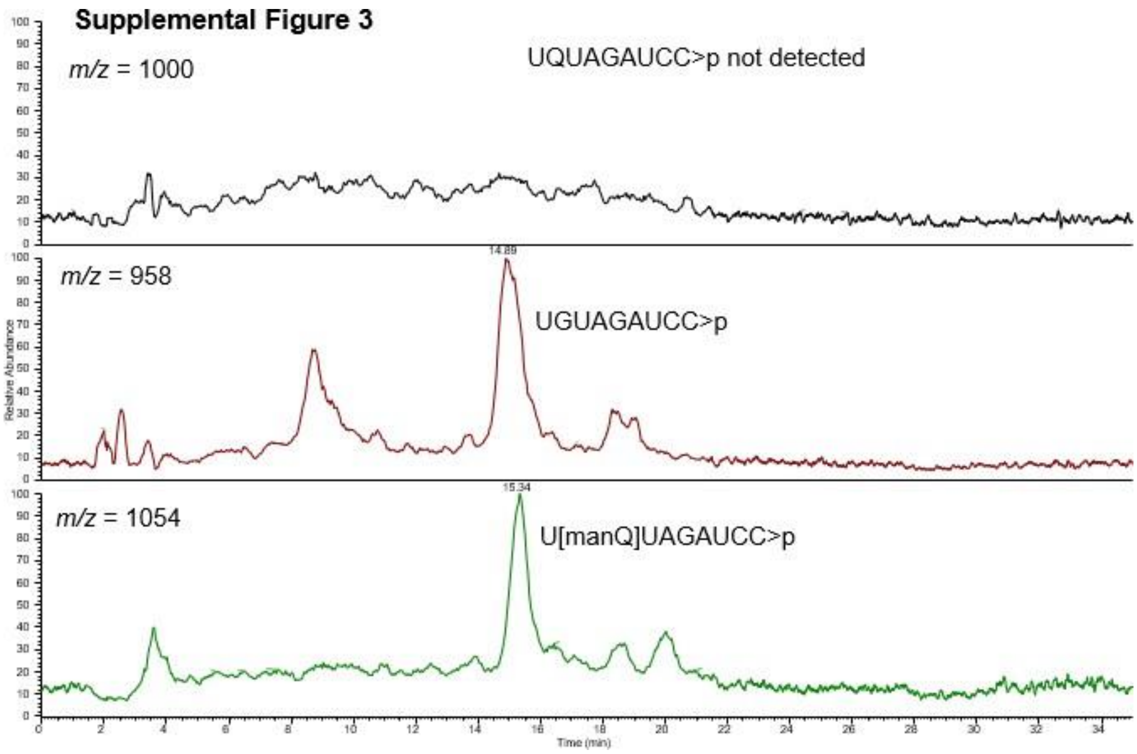

Supplemental Figure 3. XIC of whole-body Tyr anticodon oligonucleotides generated by cusativin digestion. Q was not detected at position 34 as the predicted digestion product, U[Q]UAGAUCC>p (m/z = 1000) was not detected (top). An unmodified oligonucleotide was

detected for the Tyr anticodon, UGUAGAUCC>p (m/z = 958) (middle). The position 34 modification on the Tyr anticodon is manQ and this was detected in the oligonucleotide U[manQ]UAGAUCC>p (m/z = 1054) (bottom). The Tyr anticodon sequence is unique and there were not any other tRNAs that contain this particular sequence, providing additional confidence in oligonucleotide identification.
